# A common goodness-of-fit framework for neural population models using marked point process time-rescaling

**DOI:** 10.1007/s10827-018-0698-4

**Published:** 2018-10-08

**Authors:** Long Tao, Karoline E. Weber, Kensuke Arai, Uri T. Eden

**Affiliations:** Boston University College of Arts and Sciences, 111 Cummington Mall, Boston, MA 02215 USA

**Keywords:** Neural modeling, Neural population activity, Spike trains, Goodness-of-fit, Time-rescaling, KS plots

## Abstract

A critical component of any statistical modeling procedure is the ability to assess the goodness-of-fit between a model and observed data. For spike train models of individual neurons, many goodness-of-fit measures rely on the time-rescaling theorem and assess model quality using rescaled spike times. Recently, there has been increasing interest in statistical models that describe the simultaneous spiking activity of neuron populations, either in a single brain region or across brain regions. Classically, such models have used spike sorted data to describe relationships between the identified neurons, but more recently clusterless modeling methods have been used to describe population activity using a single model. Here we develop a generalization of the time-rescaling theorem that enables comprehensive goodness-of-fit analysis for either of these classes of population models. We use the theory of marked point processes to model population spiking activity, and show that under the correct model, each spike can be rescaled individually to generate a uniformly distributed set of events in time and the space of spike marks. After rescaling, multiple well-established goodness-of-fit procedures and statistical tests are available. We demonstrate the application of these methods both to simulated data and real population spiking in rat hippocampus. We have made the MATLAB and Python code used for the analyses in this paper publicly available through our Github repository at https://github.com/Eden-Kramer-Lab/popTRT.

## Introduction

Statistical models have proven to be a powerful approach to capturing the coding properties of neural systems (Brown et al. 2004; Kass et al. 2005, 2014; Paninski et al. 2007). In addition to describing the associations between spiking activity and the biological and behavioral signals being represented, they also provide tools for model assessment and refinement. As electrophysiological experiments have become more sophisticated, incorporating simultaneous spiking data from more neurons across multiple brain areas, the focus of neural data analysis problems has begun to shift from ones that attempt to understand the tuning properties of individual neurons to ones that attempt to capture the combined structure of activity from neural populations (Georgopoulos et al. 1986; Wu et al. 2002; Pillow et al. 2008; Paninski et al. 2009; Shanechi et al. 2012). This shift has generated a need for statistical modeling and goodness-of-fit tools that can address neural coding problems at the population level.

Considering spikes as localized events in time, which are most appropriately described using the theory of point processes, has led to a class of statistical models that has been highly successful at capturing the coding properties and dynamics of individual neurons (Kass and Ventura 2001; Truccolo et al. 2005; Pillow et al. 2008). The traditional neural point process modeling framework relates the spiking activity of isolated or sorted neurons to their own recent spiking history, that of other neurons in its network, and to the behavioral and biological signals to which the neurons respond (Brown et al. 2002; Smith and Brown 2003; Truccolo et al. 2005; Deng et al. 2013; Arai and Kass 2017). Notable examples include modeling of spatial coding and movement trajectories using firing in the CA1 region in the rat hippocampus (Brown et al. 1998; Huang et al. 2009; Eden et al. 2018), as well as the neural decoding of hand velocities and collective dynamics in the primary motor cortex (Georgopoulos et al. 1986; Eden et al. 2004; Brockwell et al. 2004; Srinivasan et al. 2006). Relating population neural activity to behavior may be improved if instead of using spikes sorted according to neural identity, sorting is skipped entirely, and a joint model of behavior and features of unsorted spike waveforms across the neural population is built directly (Kloosterman et al. 2014; Deng et al. 2015; Sodkomkham et al. 2016).

Models of this type can be described using the theory of marked point process models (Daley and Vere-Jones 2003), in which each spike is associated with a random mark variable. In this case, the mark could be the full spike waveform, but is often taken instead to be some feature or low dimensional set of features related to the waveform, such as amplitude or half-width. Marked point process models can also be used to describe spiking activity from populations of sorted spikes, where the mark is often a discrete label indicating into which cluster each spike was sorted. Due to the generality of this class of marked point process models and its ability to model both sorted and unsorted population spiking data, it is of great importance that a corresponding set of tools for model assessment and validity, commonly referred to as goodness-of-fit, be developed. When properly developed and implemented, these types of model assessment metrics are helpful for determining whether a model accurately reflects the structure of a neural representation and whether the representation remains stable in the face of experimental dynamics. They can also provide a way to further refine a given model and understand the specific ways in which it may be underperforming or lacking fit.

Multiple goodness-of-fit tools have been established for point process models of individual neurons. Notably, many of these methods are based on a fundamental theoretical result known as the time-rescaling theorem (Papangelou 1972; Brown et al. 2002), which states that any point process representing a neural spike train can be rescaled based on its instantaneous spiking intensity so that it becomes a simple Poisson process with a constant spike rate. In terms of model assessment, this means that for any proposed neural spiking model, we can rescale the observed spikes according to that model and assess the goodness-of-fit between the rescaled spiking and the known properties of Poisson processes. Notably, researchers often use Kolmogorov-Smirnov (KS) plots, which compare the empirical distribution of the rescaled interspike intervals to the distribution of interspike intervals expected from a Poisson process. This is one of a range of goodness-of-fit tools made available through the time-rescaling approach.

However, with the expanding development of these new marked point process models for population data, there is a need for a corresponding development of appropriate goodness-of-fit tools that can be applied generally to these models. Gerhard et al. (2011) describe an approach based on time-rescaling multiple univariate point processes. Vere-Jones and Schoenberg (2004) prove the general time-rescaling theorem for marked-point processes, but do not develop its use for goodness-of-fit over a fixed observation interval. In this paper, we describe a new methodology that extends these approaches, based on a generalization of the time-rescaling theorem to marked point processes. We provide a heuristic proof of the theorem, and illustrate the method with simulated and real data from population spiking.

The key idea behind this generalization is to consider a marked point process model as providing a description of the spiking intensity about a neighborhood of any mark value, and to rescale each observed spike individually, based on its mark. The marked point process time-rescaling theorem then indicates that the resulting rescaled marked point process has spikes that are uniformly distributed in time and mark space, in a region that is defined by rescaling the observation interval, [0,T] across all marks. Therefore, assessment of marked point process models can be performed using goodness-of-fit techniques for uniformity of the spikes. Additionally, by taking the superposition of the rescaled spikes over all marks we obtain a univariate point process in time and a rescaled intensity. If the original marked point process model is correct, the resulting process will be an inhomogeneous Poisson process with the given intensity, allowing for the use of standard point process goodness-of-fit tools such as KS plots. In fact, this procedure allows for an extensive array of goodness-of-fit techniques.

Section 2 will provide a brief summary of point process modeling methods and the time-rescaling theorem in a single dimension for general univariate point processes, followed by a description of the approach for modeling neural populations as marked point processes. We will then describe a generalization of the time-rescaling theorem for these models, and provide a heuristic proof of the theorem. In Sections 3 and 4, we illustrate our model assessment method by simulation as well as a real-data application, respectively.

## Methods for goodness-of-fit based on the time-rescaling theorem

### The conditional intensity function and the time-rescaling theorem for univariate point processes

Define an observation interval [0, *T*] and let 0 ≤ *s*_1_ < *s*_2_ <,...,< *s*_*n*− 1_ < *s*_*n*_ ≤ *T* be a set of event (spike) times. Let *N*(*t*) be the number of spikes up to time *t*, which will increase by 1 at times when a spike occurs and will remain constant otherwise. Any point process *N*(*t*) describing neural spiking can be fully characterized by its conditional intensity function (Daley and Vere-Jones 2003)

1 $$\begin{array}{@{}rcl@{}} \lambda(t|H_{t})&=&\lim\limits_{{\Delta} \rightarrow 0} \frac{\Pr (\text{a spike in} (t,t+{\Delta}]|H_{t})}{{\Delta}} \\ &=& \lim\limits_{{\Delta} \rightarrow 0} \frac{\Pr (N(t+{\Delta}) -N(t)= 1|H_{t})}{{\Delta}} , \end{array} $$

where *H*_*t*_ = {0 ≤ *s*_1_ < *s*_2_ <,...,< *s*_*N*(*t*)_ ≤ *t*} is the history of spiking activity up to time *t*. The conditional intensity function expresses the instantaneous likelihood of observing a spike at time *t*, and implicitly defines a complete probability model for the point process. It therefore serves as the fundamental building block for constructing the likelihoods and probability distributions needed for the point process data analysis.

The basic idea of the time-rescaling theorem is to transform a general temporal point process to a constant-intensity Poisson process by rescaling the spike times.

#### **Theorem 1** (time-rescaling theorem)

*For a given point process* *N*(*t*) *with* *conditional intensity function* *λ*(*t*|*H*_*t*_) *with event (spike) times* 0 ≤ *s*_1_ < *s*_2_ <,...,< *s*_*N*(*T*)_ ≤ *T* *in an observation interval* [0, *T*]*,* *let*

2 $$ u_{j} = \int_{0}^{s_{j}} \lambda(t|H_{t}) dt,  $$

for *j* = 1,..., *N*(*T*). *Then* *u*_*j*_ *are the spike times of a homogeneous Poisson process with unit intensity rate, called the rescaled spike times*.

Note that *u*_*j*_, *j* = 1,..., *N*(*T*), will be independent, identically uniformly distributed on the observation interval [0, ${\int _{0}^{T}} \lambda (t|H_{t}) dt]$ (Ross 1996). Once a point process model is fitted, we can integrate the estimated conditional intensity between the observed spike times *s*_*j*− 1_ to *s*_*j*_ to get a set of rescaled interspike intervals, $\hat {z}_{j} = \hat {u}_{j} - \hat {u}_{j-1}$, which should be independent, and follow an exponential distribution with rate equal to 1 if the fitted model is correct. We can then use well studied methods for assessing whether the rescaled times are well fit by a unit rate Poisson process model. For example, the Kolmogorov-Smirnov (KS) plot, which plots an empirical distribution from data against a model distribution, can be used to compare the rescaled interspike intervals to the exponential distribution. Similarly an autocorrelation analysis of the rescaled interspike intervals should show no significant structure at any lag if the estimated conditional intensity from the fitted model accurately describes the spiking observations (Brown et al. 2002; Truccolo et al. 2005).

### The joint mark intensity function and the general time-rescaling theorem for marked point processes

We describe spike data from a neural population using a combination of the spike time, and another variable, **m**, called the mark, which can provide information about the spike waveform or the identity of the neuron to which that spike is associated (Kloosterman et al. 2014; Deng et al. 2015). This mark may be discrete (e.g. Neuron 1 vs Neuron 2) or continuous (e.g. spike amplitude); it may be univariate, a vector (e.g. spike amplitude from each channel in a tetrode), or even a function (e.g. a continuous waveform function). The population spiking activity is then given by the set of observations (*s*_1_, **m**_1_),(*s*_2_, **m**_2_),...,(*s*_*n*_, **m**_*n*_).

A marked point process is completely defined by its joint mark intensity function such that:

3 $$\begin{array}{@{}rcl@{}} &&\int_{M}^{} \lambda (t,\mathbf{m}|H_{t}) d \mathbf{m} \\ &=& \lim\limits_{ {\Delta} \rightarrow 0} \frac{ \Pr (\text{a spike with mark vector \(\mathbf{m}\) in \(M\) in} (t, t+ {\Delta} ]|H_{t})}{{\Delta}} ,\\ \end{array} $$

where *M* is a subset of the mark space $\mathscr{M}$ and *H*_*t*_ is the history of spiking activity, including all the marks, up to time *t*. Here *λ*(*t*, **m**|*H*_*t*_) characterizes the instantaneous likelihood of observing a spike with mark **m** at time *t*. For fixed value **m** and *t*, *λ*(*t*, **m**|*H*_*t*_) may depend on the past history of spikes with similar marks (corresponding to the intrinsic history dependence of each neuron), on the history of spikes with dissimilar marks (corresponding to functional connectivity between neurons), and on the extrinsic covariates that the neural population is encoding (for example, place and movement coding in rat hippocampus).

Taking an integral of *λ*(*t*, **m**|*H*_*t*_) over the entire mark space $\mathscr{M}$,

4 $$ {\Lambda} (t|H_{t}) = \int_{\mathscr{M}}^{} \lambda(t,\mathbf{m}|H_{t}) d \mathbf{m},  $$

gives the conditional intensity of observing a spike at time *t* regardless of the mark value. Λ(*t*|*H*_*t*_) is often called the ground intensity of the marked point process (Daley and Vere-Jones 2003).

Marked point process modeling has been successfully applied to multi-unit spiking data (Kloosterman et al. 2014; Deng et al. 2015) and to the analysis of simultaneously recorded spike events (Ba et al. 2014). While some theoretical results related to time-rescaling of the marked point processes have been developed (Vere-Jones and Schoenberg 2004), a complete goodness-of-fit paradigm for population spiking models over fixed observation intervals has yet to be established. Here, we present a general time-rescaling theorem for marked point processes observed on a finite observation interval [0, *T*], with marks that could be either continuous or discrete.

#### **Theorem 2** (General time-rescaling theorem)

*For a marked point process with observed marks* $\mathbf {m}_{i} \in \mathscr{M}$*,i* = 1,..., *N*(*T*)*, associated with the spike* *times* 0 ≤ *s*_1_ <,...,< *s*_*N*(*T*)_ ≤ *T* *and with joint* *mark intensity function* *λ*(*t*, **m**|*H*_*t*_)*.* *Let*

5 $$ \tau_{j}(\mathbf{m}) = \int_{0}^{s_{j}} \lambda(t, \mathbf{m}|H_{t}) dt, \text{ for} j = 1,...,N(T)  $$

be a set of rescaled spike times, let

6 $$ b(\mathbf{m}) = {\int_{0}^{T}} \lambda(t, \mathbf{m}|H_{t}) dt,  $$

be a mark dependent boundary based on the rescaled value of *T* for each mark, and let *R* = {(*τ*, **m**) : 0 ≤ *τ* ≤ *b*(**m**)} be a stochastic region defined by this boundary. Then the joint distribution of $\{(\tau _{j}, \mathbf {m}_{j})\}_{j = 1}^{N(T)}$ and the number of spikes in region *R* is equal to that of a homogeneous marked Poisson process with constant mark intensity equal to 1. Therefore, conditional on the boundary *b*(**m**), each (unordered) spike is independently, uniformly distributed in the region *R*.

A heuristic proof of this theorem arises from a simple change of variables. Consider the joint probability distribution of all of the spike times and marks, which is given by the product of the joint mark intensity function, $\lambda (s_{j}, \mathbf {m}_{j}|H_{s_{j}})$, at the spike locations and the exponential of the negative integral of *λ*(*t*, **m**|*H*_*t*_) over the whole time-mark space:

7 $$\begin{array}{@{}rcl@{}} & &p(\{(s_{j}, \mathbf{m}_{j}), j = 1,...,n\}, N(T)=n) \\ &=& \prod\limits_{j = 1}^{n} [\lambda(s_{j}, \mathbf{m}_{j}|H_{s_{j}})] e^{ -\int_{\mathscr{M}}^{} {\int_{0}^{T}} \lambda(t, \mathbf{m})dtd\mathbf{m}} . \end{array} $$

Note that these marked spikes completely specify the joint mark intensity (which is history dependent) everywhere in the observation interval and therefore also specify the extent of the stochastic region *R*. By the multivariate change-of-variables formula (Port 1994), the joint distribution of the rescaled times and marks is given by the expression:

8 $$\begin{array}{@{}rcl@{}} && p(\{(\tau_{j}, \mathbf{m}_{j}), j = 1,...,n\}, N(T)=n) \\ &=& p(\{(s_{j}, \mathbf{m}_{j}), j = 1,...,n\}, N(T)=n) \left| \frac{\partial \tau} {\partial s} \right|^{-1}. \end{array} $$

The elements of $\left [\frac {\partial \tau _{i}}{\partial s_{j}} \right ]$ are equal to $\lambda \left (s_{j}, \mathbf {m}_{j}| H_{s_{j}}\right )$ if *i* = *j*, and are 0 if *i* < *j*. Therefore $\left [\frac {\partial \tau }{\partial s} \right ]$ is a lower triangular matrix, and its determinant is given by the product of its diagonal terms, $|\frac {\partial \tau }{\partial s}| = \prod _{j = 1}^{n} \lambda (s_{j} , \mathbf {m}_{j} |H_{s_{j}})$, so that

9 $$\begin{array}{@{}rcl@{}} &&p(\{(\tau_{j}, \mathbf{m}_{j}), j = 1,...,n\}, N(T)=n) \\ &=& e^{ -\int_{\mathscr{M}}^{} {\int_{0}^{T}} \lambda(t, \mathbf{m})dtd\mathbf{m}} =e^{- \int_{\mathscr{M}}^{} b(\mathbf{m}) d\mathbf{m}} = e^{-|R|}, \end{array} $$

where |*R*| is the volume of region *R*. This is equivalent to the joint distribution of a marked point process with constant unit joint mark intensity over the region *R*.

We can further conclude that the number of spikes in region *R* follows a Poisson distribution with mean equal to |*R*|. Thus the conditional joint distribution of rescaled spike times given that there are *n* spikes in the region *R* is

10 $$\begin{array}{@{}rcl@{}} &&p(\{(\tau_{j}, \mathbf{m}_{j}), j = 1,...,n\}| N(T)=n) \\ &=& \frac{ p(\{(\tau_{j}, \mathbf{m}_{j}), j = 1,...,n\}, N(T)=n)}{\Pr (N(T) = n)} \\ &=& \frac{e^{-|R|}}{ |R|^{n} e^{-|R|}/n!} \\ &=& \frac{n!}{|R|^{n}}. \end{array} $$

This is exactly the joint density function of a temporally ordered set of independent uniformly distributed events in the rescaled stochastic region *R*.

Here, we presented a heuristic proof of the marked point process time-rescaling theorem based on a change-of-variables argument with the intension of providing intuition about the effect of rescaling. A complete proof requires a few additional details to ensure that the resulting process is well behaved, and more technical proofs are available in the literature (Meyer 1971; Brown and Nair 1988; Vere-Jones and Schoenberg 2004).

Based on the time-rescaling theorem result above, we can also derive the spike rate for the ground process of all the rescaled spikes across all marks.

#### **Corollary 1**

*For a rescaled, marked point process with unit joint intensity function in regionR* *as* *defined above, the (rescaled) spike times will be an inhomogeneous Poisson process with conditional intensity given by*

11 $$ \tilde{\lambda}(\tau) = \int_{\mathscr{M}}^{} \lambda_{o}(\tau, \mathbf{m}) d \mathbf{m},  $$

*where*

12 $$ \lambda_{o}(\tau, \mathbf{m}) = I_{\{(\tau, \mathbf{m}) \in R\}},  $$

*is the indicator function that specifies whether the point* (*τ*, **m**) *is in the region* *R* *or not*.

### Simulating a marked point process using the general time-rescaling theorem

The focus of the remainder of this paper is on using the generalized time-rescaling theorem to enable methods for evaluating the goodness-of-fit of a marked point process model to recorded population spiking data. Before delving into that topic, it is worth noting that the generalized time-rescaling theorem can also be used to generate simulated spiking data from any joint mark intensity function for a marked point process. This method parallels the well-known method for generating a spike train from the conditional intensity of a univariate point process using the classic time-rescaling theorem.

For a deterministic joint mark intensity function, *λ*(*t*, **m**), the simulation procedure is relatively simple. First, compute the total integrated intensity, $\boldsymbol {{\Lambda }} = \int _{\mathscr{M}}^{} {\int _{0}^{T}} \lambda (t, \mathbf {m})dt d\mathbf {m}$, and sample the total number of spikes from a Poisson distribution with parameter **Λ**. For each of these spikes, sample its location uniformly from the region enclosed by the boundary, $b(\mathbf {m}) = {\int _{0}^{T}} \lambda (t, \mathbf {m}) dt$. This can be achieved by sampling uniformly a rectangular volume encasing this region and only accepting samples that are within the boundary. If the *i*^*th*^ uniformly sampled spike has mark **m**_*i*_ and time *τ*_*i*_, compute the spike time as $s_{i} = \min \{s:\tau _{i} = {\int _{0}^{s}} \lambda (t, \mathbf {m}) dt\}$, where min gives the minimum (technically the infimum) value of *s* that satisfies thins integral. The resulting set of spike times and marks, (*s*_*i*_, **m**_*i*_) represents a sample from the marked point process with joint mark intensity *λ*(*t*, **m**).

When the joint mark intensity depends on its own history, *λ*(*t*, **m**|*H*_*t*_), the simulation procedure is slightly more complicated, as each spike can influence the future values of the joint mark intensity as well as the computations of the total integrated intensity, the boundary, and the scaling of other spikes. In this case, the above procedure can be performed iteratively. Start by initializing with a history that includes no spikes, and generate a set of rescaled spikes. Fix the earliest occurring spike, update the joint mark intensity to include this spike time, and simulate a new set of spikes from this time forward. Continue iterating, fixing the earliest new spike, until you obtain a sample of zero new spikes from the Poisson sample. This procedure will produce a sample consistent with *λ*(*t*, **m**|*H*_*t*_), where *H*_*t*_ is the history of the sampled spikes.

### Assessing model goodness-of-fit using the general time-rescaling theorem

The marked point process time-rescaling theorem establishes the joint distribution of the rescaled spikes under the assumption that the joint mark intensity model is correct. Therefore, the problem of assessing the goodness-of-fit of any proposed model can be reduced to the simpler problem of determining whether the distribution of the rescaled spike times and marks are consistent with a unit-rate marked Poisson process, or equivalently, whether the spikes occur uniformly over the region *R*.

There are a variety of well studied approaches for assessing goodness-of-fit based on this rescaled process. These multiple methods are complimentary in that one method may detect lack of fit due to particular structure in the data that may not be detected by another method. A number of these are discussed in the discussion section, but here we focus on two relatively simple approaches that are easy to interpret and highlight multiple ways in which the model may fit the data well or poorly.

The first approach is based on Pearson’s chi-square statistic. To implement this, we divide the region *R* into *M* smaller subregions, *R*_*i*_, each with volume |*R*_*i*_|, and count the number of rescaled spikes, *r*_*i*_, in each of these subregions. The test statistic is

13 $$ X^{2}=\sum\limits_{i = 1}^{M}\frac{(r_{i} - np_{i})^{2}}{np_{i}},  $$

where $p_{i}=\frac {|R_{i}|}{|R|}$ and *n* is the total number of points in *R*. We select the subregions such that *np*_*i*_ is sufficiently large (say, above 5) in each. If our marked point process model is correct and the rescaled spikes are uniform in this region, then *X*^2^ will follow a chi-square distribution with *M* − 1 degrees of freedom. We will reject the null hypothesis that the points are uniformly distributed in region *R* if $X^{2}> \chi ^{2}_{M-1,1-\alpha }$, where $\chi ^{2}_{M-1,1-\alpha }$ is the critical value of the chi-square distribution with *M* − 1 degrees at a level of significance *α*.

Another approach for assessing the goodness-of-fit for the rescaled process is based on the Kolmogorov-Smirnov (KS) plot. For a univariate (unmarked) point process, if the model is correct, the rescaled process should be a homogeneous Poisson process with interspike intervals that have independent exponential distributions with mean 1. A KS plot then simply plots the empirical cumulative distribution function (CDF) of the rescaled times against the model CDF of an exponential distribution to visualize the deviation from the 45 degree line (Johnson and Kotz 1970). For a marked point process, the set of rescaled spike times (ignoring the mark values) should be an inhomogeneous Poisson process with rate $\tilde {\lambda }(\tau )$, as defined in Section 2.2. We can therefore rescale this process one more time, based on the univariate time-rescaling theorem, construct KS plots, and make inferences from them. Additionally, we can perform KS tests to the supposed spikes over any subspace of the full mark space. That is, the set of rescaled spike time in a subspace *R*_*s*_ ⊂ *R* should be an inhomogeneous Poisson process with rate $\lambda (\tau ) = \int _{\mathscr{M}}^{} I_{(\tau , \mathbf {m}) \in R_{s}} d \mathbf {m}$. This provides additional methods for detecting model misfit around particular marks, even when the marginal spike times over the full mark space is not rejected by the full KS test.

An alternative approach for constructing KS plots is first to normalize the rescaled spike times, *τ*_*j*_, at each mark by the value of the boundary at the mark *b*(**m**). The following corollary gives the distribution of the resulting unordered rescaled spike times.

#### **Corollary 2**

*For a marked point process with observed marks* $\mathbf {m}_{i} \in \mathscr{M}$*,i* = 1,..., *N*(*T*)*, associated with the spike* *times* 0 ≤ *s*_1_ <,...,< *s*_*N*(*T*)_ ≤ *T* *and with joint mark* *intensity function* *λ*(*t*, **m**|*H*_*t*_)*. Let*

$$\tilde{\tau}_{j}(\mathbf{m}) = \frac{\tau_{j}(\mathbf{m})}{b(\mathbf{m})}= \frac{\int_{0}^{s_{j}} \lambda(t, \mathbf{m}|H_{t}) dt}{{\int_{0}^{T}} \lambda(t, \mathbf{m}|H_{t}) dt}, $$

*for j* = 1,..., *N*(*T*) *be the normalized rescaled spike times. Ignoring the mark values, these unsorted rescaled spike times* $\tilde {\tau }_{j}$ *are the event times of a homogeneous Poisson process with rate parameter* *λ* = *N*(*T*).

Note that the resulting transformed marked process is no longer a spatiotemporal homogeneous Poisson process. On the other hand, all the normalized rescaled spike times will lie in the cubic region [0,1]^*p*^, where *p* is the dimension of joint time-mark space. The KS plot would then compare the empirical CDF of the interspike intervals of these rescaled spike times against the model CDF, the exponential distribution with parameter *λ* = *N*(*T*), without the need for an additional rescaling step. This approach is a general marked point process analogue of the method described in Gerhard et al. (2011) for multiple univariate point processes.

We will demonstrate the time-rescaling theorem as well as these two goodness-of-fit approaches to simulated data in Section 3, and to real neural population spiking data recorded from a rat performing a memory-guided spatial navigation task in Section 4.

## Simulation study

We developed a set of simple simulation examples to demonstrate the process of using this general time-rescaling approach on spike train data, both for models of sorted spikes and for clusterless models of population spiking.

### Simulation study 1

The first simulation scenario comprises two neurons with spiking tuned to a single covariate, *x*_*t*_, with coordinated, history dependent firing and overlapping mark distributions. We can think of *x*_*t*_ as a one-dimensional position variable, and our neurons as place cells with distinct place fields. Each neuron has a history dependent structure leading to a brief refractory period, and neuron 2 has an excitatory influence on neuron 1 at a lag of 10 time steps.

**Fig. 1:**
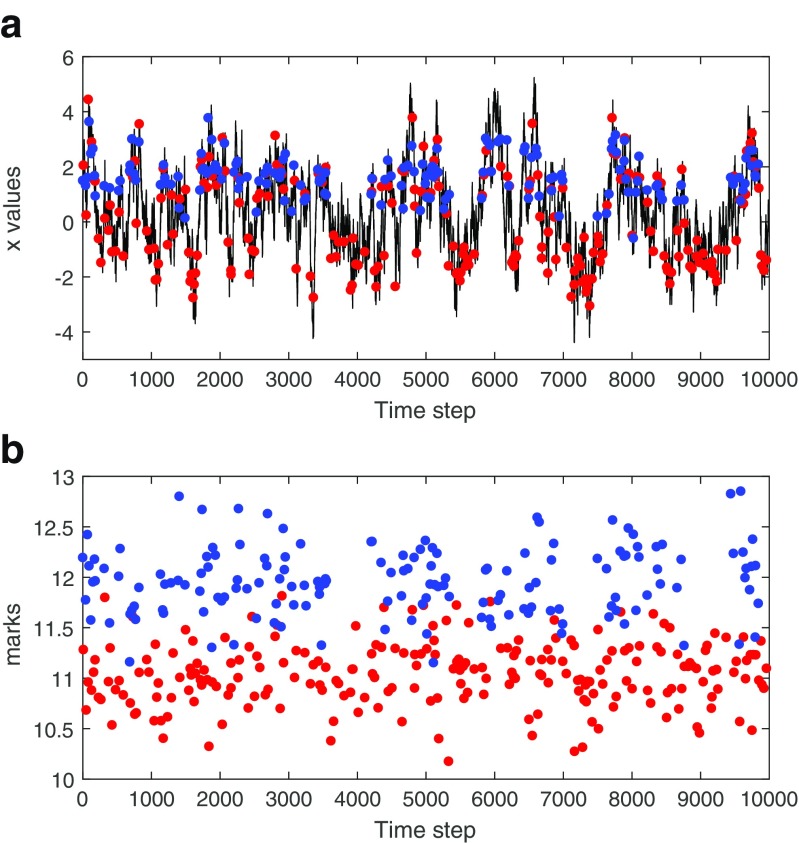
Simulated spiking from a marked point process model with joint mark intensity that depends on a state variable *x*_*t*_ defined as an AR(1) process, as defined in Eq. 15. There are 383 spikes in this example. **Panel A:** simulated x-values and spike locations in time. **Panel B:** mark values of each spike. Red and blue spike colors indicate whether a spike comes from neuron 1 or neuron 2

The position variable, *x*_*t*_, is modeled as a stationary autoregressive (AR(1)) process. Mathematically, we define the state update equation for *x*_*t*_ as:

14 $$ x_{t} = \alpha x_{t-1} + \epsilon_{t},  $$

where *α* = 0.98 and *𝜖*_*t*_ is a zero mean white noise process, with standard deviation 0.3. The top panel of Fig. 1 shows a realization of *x*_*t*_, over 10,000 time steps.

Spiking data was simulated according to a marked point process model with a joint mark intensity function with two modes, each corresponding to place fields with different locations in space and mark values. Both the spike time and mark are generated as a function of the process *x*_*t*_ and the mark can be thought of as a waveform amplitude. The two peaks are centered at 2 and − 2 in position and 11 and 12 in mark space. These peaks are each modeled as Gaussian functions with peak values of 0.15 spikes per time step and covariance matrix [0.5, 0; 0, 0.09]. This leads to moderate overlap between the peaks in the mark space (making perfect spike sorting impossible) but minimal overlap in the place coding. Finally, each neuron has a refractory period defined by the negative of a Gaussian function, centered at zero lag after a spike and with a standard deviation of 14 time steps, and neuron 2 has an excitatory influence on neuron 1 defined by a positive Gaussian function, centered at lag 10 time steps after a spike and with a standard deviation of 2 time steps.

Mathematically, the population spiking model is given by the joint mark intensity function

15 $$\begin{array}{@{}rcl@{}} \lambda(t, m) &= & [\lambda_{x1}(x_{t}) + \lambda_{E1}(H_{t})]\cdot \lambda_{H1}(H_{t}) \cdot N(m; \mu_{m1}, \sigma^{2}_{m1}) \\ &&+ \lambda_{x2}(x_{t})\cdot \lambda_{H2}(H_{t}) \cdot N(m; \mu_{m2}, \sigma^{2}_{m2}), \end{array} $$

where

$$\lambda_{x1} (x_{t}) = \exp\left[ a_{1} - \frac{(x_{t} - \mu_{x1})^{2}}{2 \sigma^{2}_{x1}}\right] $$

and

$$\lambda_{x2} (x_{t}) = \exp\left[ a_{2} - \frac{(x_{t} - \mu_{x2})^{2}}{2 \sigma^{2}_{x2}}\right] $$

represent the place fields for neurons 1 and 2 respectively,

$$\lambda_{E1}(H_{t}) = \sum_{i = 1}^{N(t^{-})} \exp \left[a_{3} - \frac{(t -s_{i} - r)^{2}}{2 {\sigma^{2}_{1}}}\right] I_{\{s_{i} \in S_{2}\}}, $$

represents the excitatory influence of neuron 2 on neuron 1,

$$\lambda_{Hj}(H_{t}) = \prod_{i = 1}^{N(t^{-})}\left[1-\exp \left[ - \frac{(t -s_{i})^{2}}{2{\sigma^{2}_{2}}}\right]\right]I_{\{s_{i} \in S_{j}\}}, $$

for *j* = 1,2 represents the refractoriness of neuron *j*, and $N(m; \mu _{mj}, \sigma ^{2}_{mj})$ expresses the normal distribution of marks for neuron *j*.

Here, *N*(*t*^−^) is the total number of spikes up to, but not including, time *t*, and for neuron index *j* = 1,2, *S*_*j*_ are the sets of spike times. The parameters *a*_*j*_ are the numeric values for the peak firing rates, the *μ*_*xj*_, *μ*_*mj*_ are centers and the $\sigma ^{2}_{xj}$, $\sigma ^{2}_{ml}$ are variances in location and mark space for these 2 place cells, and the ${\sigma ^{2}_{j}}$ is the variance of excitatory influence and refractoriness. *a*_3_ is the peak excitatory influence from neuron 2 on the firing rate of neuron 1. The numeric values for these constants used in the simulation can be found in Table 1.

**Table 1:** Simulation study model parameters

*a*_1_	*a*_2_	*a*_3_	*μ*_*x*1_	*μ*_*x*2_	*σ*_*x*1_	*σ*_*x*2_	*μ*_*m*1_	*μ*_*m*2_	*σ*_*m*1_	*σ*_*m*2_	*σ*_1_	*σ*_2_	r
$\log (0.15)$	$\log (0.15)$	$\log (0.3)$	-2	2	$\sqrt {.5}$	$\sqrt {.5}$	11	12	0.3	0.3	2	14	10

Figure 1 shows the simulated spiking from this population as a function of the simulated *x*_*t*_ trajectory. There are a total of 383 spikes in this example. In panel A, spikes are shown as a function of time and position as red and blue dots. The red and blue coloration indicate whether a spike comes from neuron 1 or neuron 2, respectively. We can see a set of red spikes that tend to occur whenever *x*_*t*_ is near -2, and a set of both blue and red spikes that occur whenever *x*_*t*_ is near 2. This is due to the place field of neuron 2 and its excitatory influence on neuron 1. Note that the purpose of this simulation is not to mimic actual place field populations accurately and find the best model to fit, but to generate data that will provide intuition and highlight the ability of the general time-rescaling theorem to assess the goodness-of-fit in data with different types of dependence structures.

Using the simulated data, we performed goodness-of-fit analysis using the time-rescaling theorem we developed above on three possible spiking models. The first uses the true model that generated the data from Eq. 15, including the correct structure for the place fields and the mark distribution, and the full history dependence capturing the refractoriness of each neuron and the excitatory influence of neuron 2 on neuron 1.

The second model uses the correct place and mark structure of the spiking, but omits the history dependent structure completely. Mathematically, this is given by the joint mark intensity function

16 $$\begin{array}{@{}rcl@{}} \lambda(t, m) &= & \lambda_{x1}(x_{t}) \cdot N(m; \mu_{m1}, \sigma^{2}_{m1}) \\ && +\lambda_{x2}(x_{t}) \cdot N(m; \mu_{m2}, \sigma^{2}_{m2}). \end{array} $$

The third model uses a crude spike sorting procedure based on whether each mark value is above or below 11.5, to fit individual intensity models for each of the two sorted neurons. Each neuron has the correct place field structure and history dependent structure, but some spikes are mis-sorted due to the overlap in the mark distribution. Mathematically, the pair of the intensity models for these neurons are given by the following equations:

$$ \lambda_{1} (t) = [\lambda_{x1}(x_{t}) + \tilde{\lambda}_{E1}(H_{t})]\cdot \tilde{\lambda}_{H1}(H_{t})  $$

and

17 $$ \lambda_{2}(t) = \lambda_{x2}(x_{t}) \cdot \tilde{\lambda}_{H2}(H_{t})  $$

where the excitatory and refractory history dependent component now use the sorted spike identities:

$$\begin{array}{@{}rcl@{}} \tilde{\lambda}_{E1}(H_{t}) &=& \sum\limits_{i = 1}^{N(t^{-})} \exp \left[a_{3} - \frac{(t -s_{i} - r)^{2}}{2{\sigma^{2}_{1}}}\right] I_{\{m_{i} > 11.5\}}, \\ \tilde{\lambda}_{H1}(H_{t}) &=& \prod_{i = 1}^{N(t^{-})}\left[1-\exp \left[ - \frac{(t -s_{i})^{2}}{2 {\sigma^{2}_{2}}}\right]\right]I_{\{m_{i} \leq 11.5\}}, \end{array} $$

and

$$\tilde{\lambda}_{H2}(H_{t}) = \prod_{i = 1}^{N(t^{-})}\left[1-\exp \left[ - \frac{(t -s_{i})^{2}}{2{\sigma^{2}_{2}}}\right]\right]I_{\{m_{i} > 11.5\}}. $$

The parameters for the centers and variances, including *μ*_*x*1_, *μ*_*x*2_, *μ*_*m*1_, *μ*_*m*2_, *σ*_*x*1_, *σ*_*x*2_, *σ*_*m*1_ and *σ*_*m*2_, were fit from the simulated data using a maximum-likelihood estimate (MLE). The excitatory influence parameter, *r*, was estimated using the method of moments from the cross-correlation coefficients. The remaining model parameters, including *a*_1_, *a*_2_, *a*_3_, *σ*_1_ and *σ*_2_, were assumed to be known.

**Fig. 2:**
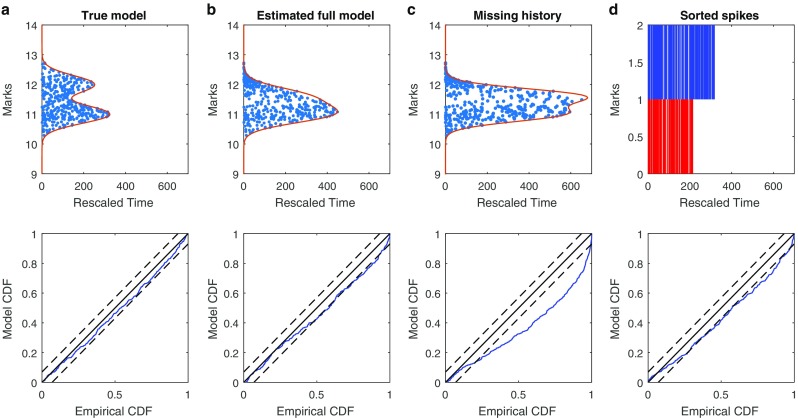
Goodness-of-fit analysis for simulated data based on candidate models: Leftmost panels (**a**) use the true model and parameter values that generated the data, including correct structure for place fields, marks, and history dependence; Middle-left panels (**b**) use the complete model with parameters estimated by maximum likelihood; Middle-right panels (**c**) use an estimated model that includes the correct structure for place fields and marks, but omits the history dependence; Rightmost panels (**d**) use an estimated model that includes correct structure for place fields and history dependence, but uses crude spike sorting rather than true mark structure. Top panels show rescaled spike times (blue dots) and observation intervals (red line) across all mark values. For spike sorted model, rescaled times for each cluster are shown. Bottom panels show KS plots based on all rescaled spike times. The true model produces rescaled spikes that are uniformly distributed in time-mark space with p-value= 0.37 for the Pearson chi-square test and a KS plot that stays within 95*%* confidence bands. The estimated true model produces rescaled spikes that are not uniformly distributed in time-mark space with p-value ≤ 10^− 5^ for the Pearson chi-square test and a KS plot that stays roughly within 95*%* confidence bands. The Pearson chi-square test for the model missing history dependence has a p-value < 10^− 5^, indicating non-uniformity of rescaled spikes. The two intensity models for the sorted spikes demonstrate lack of fit in the KS plots

Figure 2 shows the results of the time-rescaling analysis applied to each of the proposed models, using the same simulated spike data. The leftmost panels (Fig. 2a) show the goodness-of-fit assessment based on the true model and the true parameters used to generate the data given by Eq. 15. The next pair of panels (Fig. 2b) to the right show the analysis using the complete model form in Eq. 15 with parameters estimated by maximum likelihood. The next pair of panels (Fig. 2c) to the right show the goodness-of-fit for the marked point process model in Eq. 16 with correct mark and state dependence, but missing the history dependent component and with estimated parameter values. The rightmost panels (Fig. 2d) show the goodness-of-fit based on crudely sorted spikes given by the models in Eq. 17 with the correct state and history dependence structure and estimated parameters. The top panels show the rescaled spike times for each model. For the top-right panel, this is just the rescaled spikes for the two sorted neurons. For the other top panels, the rescaled spike times are given by blue dots, and the rescaled values of the end of the observation interval, *τ*(*m*, *T*), are shown as a function of *m* as a solid red line. The bottom panels show KS plots for all of the rescaled spike times under each of these models.

For the true model with the true parameter values, the value of *τ*(*m*, *T*) has local peaks around mark values of 11 and 12, corresponding to the two peaks in the joint mark-intensity function at these values. The peak around *m* = 11 is larger because of the excitatory influence in the history dependence from neuron 2 to neuron 1. Visually, the rescaled spike times appear to fill out this rescaled time-mark subspace uniformly. To perform the Chi-square test, we partitioned the mark space into a grid with *M* equal segments, and partitioned the rescaled region *R* up into *M* subregions *R*_*i*_. The number *M* was chosen such that the expected number of spikes in each subregion is above 5. We then counted the number of rescaled spikes, *r*_*i*_, in each of these subregions and calculated the Chi-square statistic by Eq. 13. The Pearson chi-square test for homogeneity of the rescaled times in this interval yields a p-value of 0.37, suggesting no clear evidence of inhomogeneity. The KS plot everywhere stays within its 95*%* significance bounds, suggesting no clear lack of fit among the full set of rescaled spike times.

The middle-left panels (Fig. 2b) show the goodness-of-fit analysis results using this same model, with parameters estimated by maximum likelihood. It is immediately evident that the quality of fit differs for the model between the true and estimated parameter values. The two clear modes in the boundary region under the true parameters are not present in the boundary using the estimated parameters, and there are evident regions where the density of the rescaled spikes in reduced. The Pearson test for uniformity now gives a p-value of *p* = 1.1 × 10^− 10^. Similarly, the KS plot now slightly departs from its 95*%* bounds, suggesting some lack of fit due to the imprecision of the parameter estimates.

For the marked point process model missing the history dependent structure (middle-right panel (Fig. 2c)), the peak around *m* = 12 is larger because *x*_*t*_ stays near the place field of neuron 2 more often than neuron 1, while the missing history dependence does not affect the intensity. By eye, it seems that the rescaled times for mark values below 11.5 occur more densely than those for mark values above 11.5. This is borne out by the Pearson chi-square test (*p* < 10^− 10^), which suggests inhomogeneity on the rescaled times, and therefore lack of fit between the model and the original spike data. The lack of fit is also visible in the KS plot, where the observed rescaled interspike intervals are consistently significantly larger than the model estimates.

The panel on the top right shows the rescaled times based on two sorted clusters. As a population model, this could be considered as a marked point process where the marks represent the cluster assignment. In that case, rescaling each spike according to its mark is equivalent to rescaling based on the intensity for whichever neuron the spike is clustered into. Missorted spikes therefore tend to be incorrectly scaled, leading to lack of fit, as observed through the KS plot.

### Simulation study 2

We performed a second simulation to illustrate how the KS plot and chi-square test highlight different aspects of the goodness-of-fit. We consider again the same two neurons tuned to a single covariate *x*_*t*_, and remove the history dependence of spiking, so that two neurons are simply inhomogeneous Poisson spiking units. In this case, the true joint mark intensity model, from which we generate the data, has the same form as Eq. 16, with parameters given in Table 1.

**Fig. 3:**
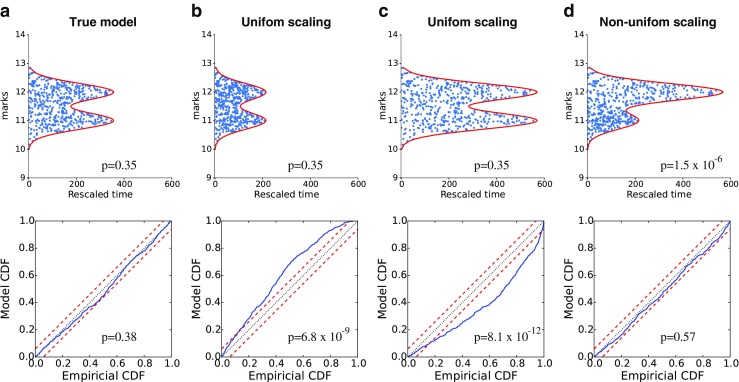
Goodness-of-fit analysis for simulated data based on four candidate models: The top panels show the rescaled spikes (blue) and region, R (red), and the p-values for the chi-square test; the lower panels show KS plots and corresponding p-values. The models are (left panel) true model, (next two panels) true model *λ*(*x*_*t*_, *m*) scaled uniformly by 0.56 and 1.6, and (right panel) true model whose components *a*_*c*_, *c* = 1,2 scaled separately by 0.56 and 1.6, respectively. The last model roughly preserves overall firing rate. KS plots detect the correctness of the firing rate irrespective of mark value, while the Pearson chi-square test characterizes how well the model captures mark structure of the joint mark intensity, which explains why the middle two wrong models still pass the Pearson chi-square test, while the last model passes the KS test

Figure 3 shows a goodness-of-fit analysis on the resulting data for four different candidate models we propose, Fig. 3a, the true model, Fig. 3b and c, two models whose *λ*(*t*, *m*) are uniformly scaled by 0.56 and 1.6, respectively, and Fig. 3d, a non-uniformly scaled model, with *a*_*c*_ (from Table 1) scaled separately by 0.56 and 1.6, for *c* = 1,2. Rescaling of the spikes according to the true model *λ*(*t*, *m*) produces good fits according to both tests, while the uniformly scaled candidate models pass the Pearson chi-square test with p-value 0.35, but the KS plot are far from being in the 95% confidence bounds. The deviation direction from the 45 degree line can be used to determine that the misspecified models underestimate and overestimate the intensity, respectively. Utilizing 523 spikes, the non-uniformly scaled candidate model, where each neuron has been scaled separately while keeping the overall firing rate close to that generated by the true model, passes the KS test, but the chi-square p-value is very small at 1.5 × 10^− 6^, highlighting the complementarity of the tests.

While the overall pattern of rescaled interspike intervals doesn’t show lack of fit, the rescaled spikes are more concentrated at low mark values and less concentrated at high mark values. This example illustrates the importance of having multiple goodness-of-fit approaches to characterize different features of the data that may be captured or misspecified by a model. In this example both the KS analysis and the Pearson chi-square test are enabled by time-rescaling of the marked point process.

### Simulation study 3: power analysis

In this subsection, we perform a third simulation to illustrate how the sample size *N*(*T*) affects the statistical power for chi-square and KS tests in each of our simulation models. We consider again the same two neurons with spiking tuned to a single covariates, *x*_*t*_, with history dependent firing and overlapping mark distributions, as described in Eq. 15. In this case we compare the ability to detect model misfit in three models, the true model that generated the data, the misspecified model that is missing history dependence, and the crude sorted spike model.

**Fig. 4:**
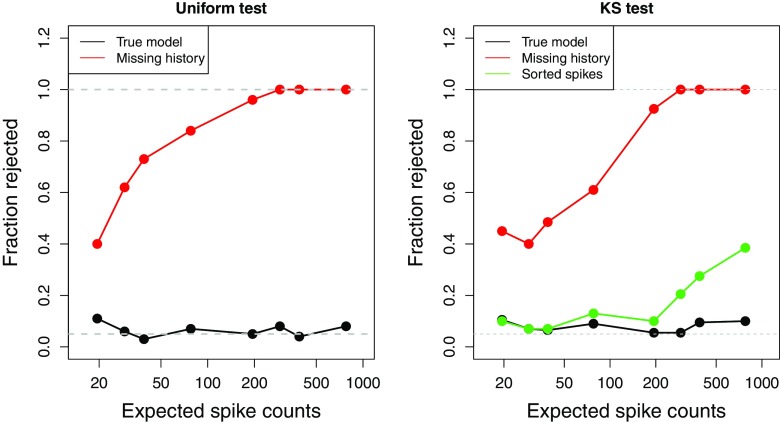
Power analysis: sample size effect Chi-square and KS test The effect of the expected spike count on the rejection rate of 100 realizations of the Pearson uniformity (left panel) and KS (right panel) tests for three different models, the true model (black line), the missing history model (red line), and the sorted spike model (blue line). The gray dotted lines indicate the significance level of 0.05 and the power = 1 value

We simulated spike trains with increasing duration ranging from .5 to 20 seconds, and performed a goodness-of-fit analysis using the time-rescaling theorem and Pearson and KS tests we described above on these three possible spiking models. We repeated each simulation 100 times and recorded the resulting Pearson uniformity test and KS test p-values. Figure 4 shows the power as a function of the expected number of spikes on the rejection rate of Pearson (left panel) and KS (right panel) tests for the three different models, the true model (black), the missing history model (red), and the sorted spike model (blue). The rejection rate is calculated as the proportion of p-values smaller than a significance level of 0.05.

From both panels, we see that when the expected number of spikes is small, both the Pearson uniformity test and KS test reject the hypothesis of a well-fit model a relatively small fraction of the time. For example, as seen in the left panel of Fig. 4, the rejected fraction of simulations using the Pearson uniformity test for the misspecified missing history model is around 0.4 when the expected number of spike is about 20, and increases to 1 as the expected spike count increases to 800. We can also see similar trend for the KS test for both the missing history model and the sorted spike model. Note that the rejection rate of KS test for the sorted spike model increases more slowly than that of the missing history model, due to the misspecification of the latter. Also, as the expected spike count becomes large, both tests will reject with a probability approaching 1.

## Data analysis

**Fig. 5:**
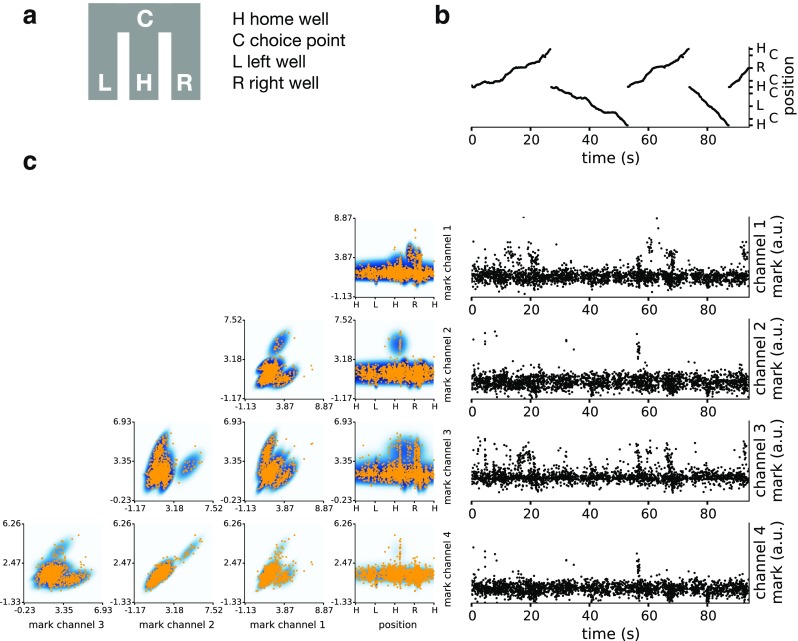
Unsorted spikes and their marks from rat CA3 as it traverses a W-shaped maze, and fitted mark intensity function: **a** Schematic of the maze. There are 4 landmarks, the home well H, the choice point C and the left L and right R reward wells. **b** Timing and location of all observed spikes in the 1-dimensional position representation. **c** Left, the fitted joint mark intensity function and spikes, shown in all combinations of 2-dimensional projections. Orange dots are spikes, and the darker color shows higher intensity. Right, timing and mark (spike amplitude) in each of the tetrode channels of observed spikes. The clustering of spikes in time is a consequence of place-specific firing

**Fig. 6:**
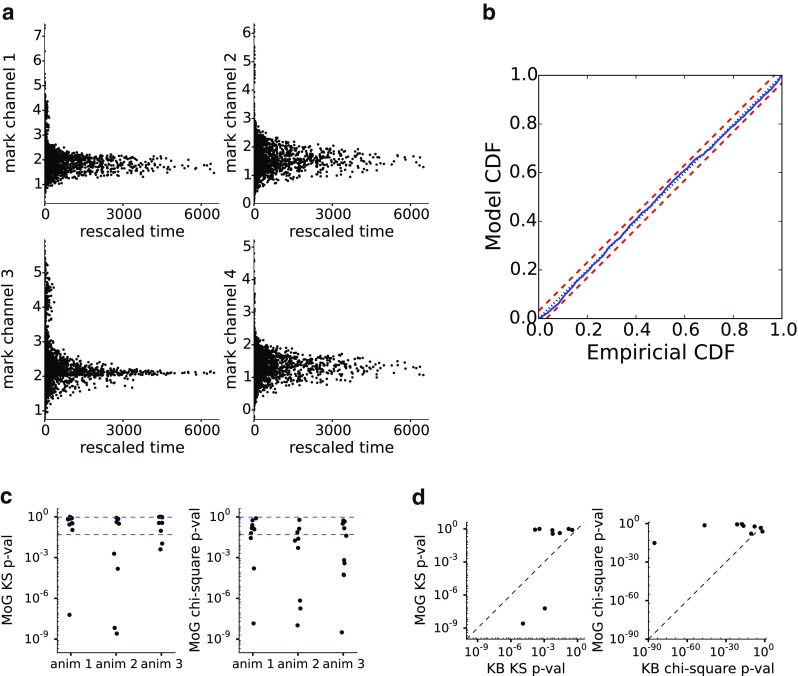
Rescaling analysis for unsorted spikes and their marks from rat CA3: **a** Time-rescaled spikes in each of the 4 tetrode channels. The same spikes appear in each of the 4 panels at the same rescaled time, but at different mark values. **b** The corresponding KS plot. The p-values for the KS test and Pearson chi-square test are = 0.81 and = 0.25, respectively. **c** Summary from 10 tetrodes each from 3 animals of p-values of the KS and Pearson chi-square tests (13/30 passed both with p-values > 0.05 for both tests) of the MoG fit of the CA1 or CA3 spiking activity. **d** A comparison of goodness of fit of MoG and KB models for the spiking activity. 3 tetrodes from each animal were chosen for MoG and KB goodness-of-fit comparison

We analyzed recordings from tetrodes placed in the CA1 and CA3 regions of hippocampus of 3 rats traversing a W-shaped environment, performing a continuous alternation task. Spikes were detected offline by choosing events whose peak-to-peak amplitudes were above a 40*μV* threshold in at least one of the channels. For each spike, the peak amplitudes across each electrode channel were used as a 4-dimensional mark. Some spikes with lower amplitude peaks may include events whose origin may not be from well-isolated neurons sought in traditional spike-sorting, and may well simply be electrical noise. These spikes are referred to as ”hash spikes”, and exist on a continuum extending below the single channel threshold often used for spike detection. In our clusterless population model, we include these hash spikes. We model the joint mark intensity using a mixture of Gaussians (MoG).

18 $$\begin{array}{@{}rcl@{}} &&\lambda(t, \mathbf{m}) = \\ &&\sum\limits_{c = 1}^{M} \lambda_{c} \exp\left[-\frac{(x_{t} - f_{c})^{2}}{2{\sigma^{2}_{c}}}- \frac{(\mathbf{m}-\boldsymbol{\mu}_{c})^{T} {\Sigma}_{c}(\mathbf{m}-\boldsymbol{\mu}_{c})}{2}\right] \end{array} $$

where *M* is the number of Gaussian components, *x*_*t*_ is the position of the animal, **m** is the four-dimensional mark vector, and *f*_*c*_, ${\sigma ^{2}_{c}}$ and ***μ***_*c*_, Σ_*c*_ are the means and covariances in position and mark spaces, respectively. Model parameters were estimated marginally, using the empirical median of the Gibbs samples (Geman and Geman 1984; Gelfand and Smith 1990) for each component as an estimator. We note that the fixed *M* is estimated at the start of the Gibbs sampling procedure, and is not the number of putative neurons that a traditional spike sorting would estimate, but is conceptually the sum over all neurons of the number of place fields that each neuron has. For the tetrode shown in Fig. 5, *M* = 31, and for the summary in Fig. 6c of the 30 tetrodes, *M* ranged from 15 to 40.

Figure 5 shows the mark data from a single example tetrode from animal 1. Figure 5b shows the time and position of occurrence of unsorted spikes, tracing out the path a rat traveled in the maze. Figure 5c, right, shows the mark value of each spike, the spike peak amplitude on each channel, as a function of time. Spikes are seen to occur preferentially at certain times, indicating place specific firing from one or more neurons. Figure 5c, left, shows the 5-dimensional joint mark intensity function, estimated based on Eq. 18, displayed two dimensions at a time. The rightmost column shows the place-specific firing structure, while the rest of the rows show various projections of the spike waveform features familiar to practitioners of manual spike sorting. Figure 6 shows the results of a time-rescaling analysis of the joint mark intensity function. Figure 6a shows the rescaled times and marks for each channel using the estimated joint mark intensity in Fig. 5c. Here, we do not expect the time-rescaled spikes to appear uniform for each 2D projection, since the other mark dimensions on which the rescaling depends have been collapsed, leading to high density at shorter times. The KS plot in Fig. 6b shows that the rescaled spikes stay in the 95% confidence bounds (KS test p-value = 0.81), and for the accompanying chi-square test for uniformity, we obtained a p-value of 0.25, suggesting that these tests do not identify substantial lack of fit in this model.

Figure 6c shows a summary of p-values of the KS and chi-squared tests for the MoG model fits. In about half the cases, the MoG model produced good fits to the data (13/30 passed both KS test and chi-squared tests with p-values > 0.05). We further utilized the GoF test to compare the MoG model to the kernel-based (KB) model described in detail in Deng et al. (2015). The KB model places a Gaussian kernel around the position and mark of each spike, whose bandwidth (BW) parameters {*B*_*x*_, *B*_*m*_, *b*_*x*_} can be varied to use more or less smoothing over the position and mark space. Under this model, the joint mark intensity function is estimated from *N* spikes observed at times {*s*_1_,...*s*_*N*_} as

19 $$ \lambda(x_{t}, \mathbf{m}) = \frac{ {\sum_{n}^{N}} K_{s}(x_{t} - x_{s_{n}})K_{m}(\mathbf{m} - \mathbf{m}_{n})} {{\Delta} t {\sum_{i}^{T}} K_{o}(x_{t} - x_{i})} $$

where Δ*t* is the bin size of the discretized time steps of the data, *T* is the total number of time bins, and the kernels are $K_{o}(x) = \frac {1}{\sqrt {2\pi {b_{x}^{2}}}}\exp \left [-\frac {x^{2}}{2{b_{x}^{2}}}\right ]$, $K_{s}(x) = \frac {1}{\sqrt {2\pi {B_{x}^{2}}}}\exp \left [-\frac {x^{2}}{2{B_{x}^{2}}}\right ]$ and $K_{m}(\mathbf {m}) = \left (\frac {1}{\sqrt {2\pi {B_{m}^{2}}}}\right )^{K}\exp \left [-\frac {\mathbf {m} \cdot \mathbf {m}}{2{B_{m}^{2}}}\right ]$. BW parameters were selected by maximizing the leave-one-out cross-validated likelihood of the observed spike data (Prerau and Eden 2011). Figure 6d shows a comparison, for 9 of these tetrodes, between the fit of this MoG model to the KB model. Many tetrodes land in the upper-right corner, where both MoG and KB models provide good fit. However, these points tend to be above the 45 degree line, suggesting improved generalizability of the MoG model.

## Discussion

### General comments

In this paper, we developed a general toolbox for assessing statistical models of neural populations based on a generalization of the time-rescaling theorem. Given technological advances in neural data acquisition, experiments involving multiple electrodes have now become standard in the practice of neuroscience, making these neural population models of great interest. Understanding these network structures sheds light on how groups of neurons interact with, react and respond to one another and help define possible functions of regions of the brain (Chen et al. 2011; Macke et al. 2011). In addition, the prevalence of multiunit data has brought into question the necessity of spike sorting in every neural population analysis. While many population analyses begin with a spike sorting step and a characterization of the receptive field properties of each sorted neuron, multiple recent experiments have explored the power of clusterless population models (Kloosterman et al. 2014; Deng et al. 2015). Therefore it is valuable to have goodness-of-fit tools that can apply equivalently to both sorted and clusterless population models. A fundamental challenge in assessing the goodness-of-fit of models of spiking systems is that the timing of each spike has its own distribution, based on many factors that can include coding of dynamic biological and behavioral variables, past spiking history, network effects, and adaptation. The time-rescaling theorem allows us to take any candidate model, and all the dependence structures it describes, and rescale the spikes in such a way that, if the model is correct, they should become samples from a simple uniform distribution. We can then use well-established methods for assessing uniformity to assess the quality of the original model used for rescaling. Furthermore, by taking only the rescaled spike times and disregarding the marks, we can generate a new univariate spike train and use the many existing goodness-of-fit tools for individual spike trains to assess the quality of the joint mark intensity model. An important feature of this general time-rescaling theorem is that not only are the spike times rescaled, but the observation interval [0, *T*] is also rescaled for each possible mark value. Since a joint mark intensity model can depend on other stochastic processes (such as its own history or the biological and behavioral variables encoded by the population), the intensity is itself a stochastic process, and therefore the rescaled observation region is also stochastic. Therefore, the assessment of uniformity is based both on the rescaled spike times and the rescaled region.

### Simulation and data analyses

We illustrated our approach via a series of simulation analyses as well as an application involving place cell spiking activity from the CA3 region of the hippocampus in a rat performing memory guided navigation task on a W-shaped maze. In the first simulation, we implemented the goodness-of-fit tests in three different model fits: the true model, a model intentionally missing history-dependence, and a model for which the mark corresponds to one of two labels given by spike sorting. As expected, the results indicated proper fit with the true model, and a lack-of-fit in both the model missing history dependence as well as the sorted model. This demonstrated the ability of the approach to discern different reasons for lack of fit. Importantly, we could assess the quality of fit for both sorted and clusterless spiking models and determine the degree to which sorting affected the model fit. In our second simulation, we demonstrated that distinct goodness-of-fit measures, both based on the same time-rescaling approach, could be used to determine different aspects of the model fit quality. Incorrectly scaling the intensity uniformly over all marks led to lack of fit evidenced by the KS plot but not the assessment of uniformity; differentially scaling subsets of mark values, as might occur with a model that misspecified the receptive fields of particular neurons, led to lack of fit evidenced by lack of uniformity.

In our real-data example, we used a 4-dimensional mark representing the waveform peak amplitudes across a tetrode to exhibit the ability to generalize to more complicated mark spaces. The fit of a Gaussian mixture model with no history dependence captured much of the temporal structure, as evidenced by the KS plot, but perhaps fit the spikes in some mark regions better than others, as suggested by the analysis of uniformity in disjoint subsets of the mark space. A follow-up analysis, suggested that the model may not be capturing the hash spikes as well as the higher amplitude spikes.

### Extensions and limitations

In this paper we focused on two goodness-of fit measures that could be applied to the rescaled spike times and marks, a Pearson chi-square test comparing the expected and observed number of spikes in subsets of the rescaled observation region, and a KS plot analysis based on rescaling the rescaled spike times again based on the expected rescaled spiking rate. However, there are a variety of other goodness-of-fit tools available after rescaling that could also be used, either instead of, or to compliment these analyses. For example, a well-studied statistical approach for assessing uniformity is based on Ripley’s *K*-function (Ripley 1977). This function, *K*(*x*, *r*), is defined as the expected number of points within a ball *b*(*r*) with radius *r* centered at *x*. For uniform rescaled spikes, this function should grow as *r*^*d*^, where *d* is the dimension of the mark-time space. We can compute the empirical *K* function, $\hat K(r)=\frac {1}{n}\sum _{i\neq j}I_{d_{ij}}<r/n$, where *d*_*ij*_ is the Euclidean distance between rescaled spikes *i* and *j*, and $I_{d_{ij}}<r$ is equal to 1 if that distance is less than *r*, and otherwise 0. We can then compare the empirical *K* function to the theoretical one under a uniform model to assess the quality of our original model. We can also construct confidence intervals for the estimated function and compute a corresponding p-value via Monte Carlo simulations (Baddeley et al. 2005). A variety of other well-documented and tested methods are also available (Petrie and Willemain 2013) and could be used interchangeably with those we specifically mention in this paper. A few examples include those that perform a two-sample test on a subsample of points in a high-density region and a subsample in a low-density region (Jain et al. 2002), or those that consider the distribution of distances from points to the boundary of support, both in the case of known support (Berrendero et al. 2006) and unknown support (Berrendero et al. 2012).

It is important to note that no single goodness-of-fit technique can demonstrate that a model completely captures the statistical structure present in a data. Thorough model assessment requires applying multiple goodness-of-fit tools to characterize the ways in which a model fails to capture different features of the data. Here, we focus on goodness-of-fit approaches that are enabled by time-rescaling, which have been used extensively for models of sorted spike train data. These approaches, along with complementary methods, such as those based on the model deviance, cross-validation, residual analysis, and auto and cross-correlation estimation, provide a powerful toolbox for model assessment (Ogata 1988; Truccolo et al. 2005; Kass et al. 2014).

There are a number of extensions and avenues for future exploration for this goodness-of-fit framework. In the simulations, we provided examples of how assessments based on time-rescaling could be used to help identify areas of lack of fit, and to suggest refinements to population spiking models. The ways in which different measures might be used for model refinement should be explored in more detail, and specific recommendations could be made about the best measures to use to identify particular features that should be added or altered in a model. Also, in our examples, we limit the standard point process goodness-of-fit analysis to KS plots but with the appropriate adaptations and generalizations, one could also employ other common techniques such as the QQ plot, autocorrelations of rescaled wait times, or a Fano Factor analysis to assess dispersion.

Another possible extension might focus on mark rescaling rather than time-rescaling. We could retain the observed times of each spike, and modify each spike mark to produce uniform spikes over a stochastic region with a fixed temporal extent, but random mark boundaries. For a one-dimensional mark, this could be achieved by replacing the mark of the *i*th spike, *m*_*i*_, with the integral $\int _{0}^{m_{i}}\lambda (t_{i},m)dm$ (Merzbach and Nualart 1986). An advantage of such an approach would be that the spike times would remain the same and be interpretable. For example a cluster of points at a particular time point might suggest model lack of fit specific to that time. However, since the temporal pattern of spikes would be unchanged, it would still retain all the temporal dependence structure in the original data. Additionally, how to best rescale in general mark spaces is still unknown.

Additional research could also be done on improving the computational burden of these methods in high dimensional mark spaces. While the rescaling of times is based only on the number of spikes, not the dimensionality of the marks, the computation of the boundary of the stochastic region will grow in complexity with the mark dimension, and it can be challening to determine which model features lead to lack-of-fit. There may be multiple ways to deal with this, including methods of efficiently approximating the boundary assuming smoothness of the intensity, or goodness-of-fit measures that are less sensitive or do not require direct knowledge of the full boundary.

With this method, we provide model assessment tools that can be used appropriately for both population models and sorted models and and help collect more detailed information on their respective fits. In this way, researchers can better understand the advantages and disadvantages posed by population and single-unit modeling. Ultimately, this could provide significant insights into the question of when neural network structures can be better understood with spike sorting or direct ensemble modeling. Additionally, as experiments head in more complex directions and datasets become richer, modeling methods will need to improve and develop alongside them. For researchers to maintain confidence in any conclusions drawn from the application of a particular modeling approach, a corresponding goodness-of-fit toolset is essential. Here, we present a general goodness-of-fit approach that can assess and indicate areas of lack-of-fit for a wide variety of population spiking models, enabling researchers to gain more understanding and insight into the increasingly complex data structures being made available in neuroscience. We have made the MATLAB and Python code used for the analyses in this paper publicly available through our Github repository at We have made the MATLAB and Python code used for the analyses in this paper publicly available through our Github repository at https://github.com/Eden-Kramer-Lab/popTRT.
